# Finite volume analysis of temperature effects induced by active MRI implants: 2. Defects on active MRI implants causing hot spots

**DOI:** 10.1186/1475-925X-5-35

**Published:** 2006-05-26

**Authors:** Martin HJ Busch, Wolfgang Vollmann, Dietrich HW Grönemeyer

**Affiliations:** 1 Grönemeyer Institute for Microtherapy, University of Witten/Herdecke, Universitätsstr. 142, D-44799 Bochum, Germany; 2 Department of mathematics, physics and chemistry, TFH University of Applied Sciences, Luxemburger Straße 10, D-13353 Berlin, Germany

## Abstract

**Background:**

Active magnetic resonance imaging implants, for example stents, stent grafts or vena cava filters, are constructed as wireless inductively coupled transmit and receive coils. They are built as a resonator tuned to the Larmor frequency of a magnetic resonance system. The resonator can be added to or incorporated within the implant. This technology can counteract the shielding caused by eddy currents inside the metallic implant structure. This may allow getting diagnostic information of the implant lumen (in stent stenosis or thrombosis for example). The electro magnetic rf-pulses during magnetic resonance imaging induce a current in the circuit path of the resonator. A by material fatigue provoked partial rupture of the circuit path or a broken wire with touching surfaces can set up a relatively high resistance on a very short distance, which may behave as a point-like power source, a hot spot, inside the body part the resonator is implanted to. This local power loss inside a small volume can reach ¼ of the total power loss of the intact resonating circuit, which itself is proportional to the product of the resonator volume and the quality factor and depends as well from the orientation of the resonator with respect to the main magnetic field and the imaging sequence the resonator is exposed to.

**Methods:**

First an analytical solution of a hot spot for thermal equilibrium is described. This analytical solution with a definite hot spot power loss represents the worst case scenario for thermal equilibrium inside a homogeneous medium without cooling effects. Starting with this worst case assumptions additional conditions are considered in a numerical simulation, which are more realistic and may make the results less critical. The analytical solution as well as the numerical simulations use the experimental experience of the maximum hot spot power loss of implanted resonators with a definite volume during magnetic resonance imaging investigations. The finite volume analysis calculates the time developing temperature maps for the model of a broken linear metallic wire embedded in tissue. Half of the total hot spot power loss is assumed to diffuse into both wire parts at the location of a defect. The energy is distributed from there by heat conduction. Additionally the effect of blood perfusion and blood flow is respected in some simulations because the simultaneous appearance of all worst case conditions, especially the absence of blood perfusion and blood flow near the hot spot, is very unlikely for vessel implants.

**Results:**

The analytical solution as worst case scenario as well as the finite volume analysis for near worst case situations show not negligible volumes with critical temperature increases for part of the modeled hot spot situations. MR investigations with a high rf-pulse density lasting below a minute can establish volumes of several cubic millimeters with temperature increases high enough to start cell destruction. Longer exposure times can involve volumes larger than 100 mm^3^. Even temperature increases in the range of thermal ablation are reached for substantial volumes. MR sequence exposure time and hot spot power loss are the primary factors influencing the volume with critical temperature increases. Wire radius, wire material as well as the physiological parameters blood perfusion and blood flow inside larger vessels reduce the volume with critical temperature increases, but do not exclude a volume with critical tissue heating for resonators with a large product of resonator volume and quality factor.

**Conclusion:**

The worst case scenario assumes thermal equilibrium for a hot spot embedded in homogeneous tissue without any cooling due to blood perfusion or flow. The finite volume analysis can calculate the results for near and not close to worst case conditions. For both cases a substantial volume can reach a critical temperature increase in a short time. The analytical solution, as absolute worst case, points out that resonators with a small product of inductance volume and quality factor (Q V_ind _< 2 cm^3^) are definitely save. Stents for coronary vessels or resonators used as tracking devices for interventional procedures therefore have no risk of high temperature increases. The finite volume analysis shows for sure that also conditions not close to the worst case reach physiologically critical temperature increases for implants with a large product of inductance volume and quality factor (Q V_ind _> 10 cm^3^). Such resonators exclude patients from exactly the MRI investigation these devices are made for.

## Background

Metallic implants often cause distortions inside magnetic resonance images. These effects arise either from the different susceptibility of tissue and metal, disturbing the gradient for spatial encoding, or from induced eddy currents on the metallic implant structure forming a Faraday cage [1-4]. An advantageous solution to reduce the eddy current shielding is to amplify the transmitted signal to and the detected signal from the spin ensemble inside the Faraday cage. A local amplification is possible by using resonators tuned to the Larmor frequency of a specific "Magnetic Resonance Imaging" (MRI) field strength [5-11]. These resonators operate as inductively coupled transmit and receive coils and can be integrated into the metallic structure of the implant itself or added around/on the normal implant structure.

This technology has the great advantage of amplifying the signal only, where it is needed, i. e. inside the Faraday cage. The signal or contrast behavior of the rest of the image plane (volume) is unaffected by these devices. Up to now, active MRI implants have not been tested in clinical trials, but active MRI stents have been investigated in rabbits [9,10]. Examples of MRI images with active implants are also shown in [12], whereas pictures of implant prototypes can be found in [11]. These prototypes are the basis of this publication. In case of a breakthrough of this technology, not only active MRI stents, but also larger implants like aortic stent grafts or vena cava filters are candidates for this technology.

The resonator feature adds local power losses during spin ensemble excitations inside such devices. This investigation uses the known maximum additional total power loss of the entire LC-circuit [11,12] and the known maximum hot spot power loss for defects on the electrical paths [12] to calculate temperature maps around hot spots. A defect like a rupture or a partial rupture on the electric path of active implants can be provoked by fatigue of material after a long implantation time with perpetually changing forces and permanent movements of the implant struts due to the cardiac cycle [13,14]. An analytical solution is available for thermal equilibrium and a hot spot inside a homogeneous medium, like tissue. This analytical solution is the absolute worst case, but overestimates the real situation. Firstly no imaging sequence lasts long enough to reach thermal equilibrium and secondly the metallic mesh of an active implant distributes the power loss P_hs _of a hot spot more efficiently than a pure tissue surrounding does. A finite volume analysis respects these situations more precisely and can investigate, whether failures of such resonators can cause unsafe conditions during MRI acquisitions even with some additional temperature reduction mechanisms. A robust and easy-to-implement algorithm is used for the risk analysis, because this simulation does not have to predict exact temperature increases, contrary to planning algorithms for therapies like hyperthermia or thermal ablations. Instead the analytical solution gives the principal risk for the worst case. The finite volume calculations should evaluate, if the risk also exists using a metallic wire inside homogeneous tissue and even with cooling due to blood perfusion superior to the physiological values and blood flow. Danger in our understanding means, that a substantial part of tissue volume is heated to a temperature, which can induce cell death. The simulations calculate temperature maps developing in time around a defect. From these temperature maps a critical volume with temperatures exceeding a critical value can be calculated. In order to increase the speed of the calculation the finite volume simulation assumes a cylindrical symmetry associated with a linear wire. This involves, that the hot spot power loss is split into two equal parts, which diffuse into both of the assumed fracture surfaces of the metallic wire. The further heat distribution is assumed to arise from heat conduction only, or from heat conduction and blood perfusion – implementing an algorithm based on the idea of Pennes equation [15,16] – or heat conduction, blood perfusion and blood flow – modeled as energy sink – near the hot spot. All simulations are only theoretical, but the total power loss of an intact active resonator was verified experimentally in a previous investigation [11] as a basis for this theoretical calculations.

## Methods

### Theory

#### Power loss of resonator

The total power loss *P*_loss _[W] of a resonator with his axis aligned perpendicular to the main magnetic field and exposed to a series of identical excitation pulses of an MRI investigation is given by [11,12]



where c_dc _and c_pwm _are dimensionless factors described further below, *ω*_0 _[rad/s] is the angular resonance frequency, μ_0 _[Vs/(Am)] is the permeability of vacuum, *Q *is the quality factor of resonance circuit inside tissue, *B*_1 _[T] is the amplitude or magnitude of the magnetic field established by a linear or respectively circular polarized transmit coil of the MR system, *B*_ind_= *B*_1 _*Q *is the magnetic field inside the inductance of the resonator and *V*_ind _[m^3^] is the volume of the resonators inductance. In this investigation the inductance volume and the implant volume are assumed to be equal. The factor c_dc _is the duty cycle of pulsed MR sequences and equals the ratio of the duration "rf excitation on" during the total acquisition time and the total acquisition time itself. c_pwm _describes the pulse waveform modulation and is the ratio between the energy of one excitation pulse with a maximum amplitude/magnitude *A *and the energy of a rectangular excitation pulse with same length and identical amplitude/magnitude *A*. A detailed derivation of Eq. (1) is outlined in [11,12], where also some MR image examples as well as some experimental prototypes are shown.

For a specific sequence on an MRI system with an angular resonance frequency *ω*_0_, the additional absorbed power is proportional to the product *V*_ind _*Q *(Eq. 1) of the inductance volume *V*_ind _and the quality factor *Q*. The proportionality to *Q *is surprising. The usual inverse proportionality of the power loss to the quality factor *Q *is confirmed by examining the power loss with respect to the magnetic field *B*_ind _established inside the inductance volume (*B*_ind _= *QB*_1_). For safety considerations the proportionality to the volume is important, which points out the significance of the implant size.

#### Maximum power losses of hot spots caused by fatigue fractures

The struts of stents, stent grafts or vena cava filters implanted in human vessels are exposed to perpetual changing forces and permanent movements, which can cause fatigue fractures [13,14]. A partial break in the conductor or a rupture, which is bridged by a loose contact between the broken ends with a very small contact area, may cause a hot spot, a small volume with a high power loss density. This conducting path with a high resistance can convert a substantial part of the total power loss to heat inside a point-like volume. The defect adds an additional serial resistance *R*_hs _to the overall resistance *R*_ov _of the intact resonator and reduces the quality factor according to *Q *= *ω*_0_*L*/*R*, where *L *[H] is the inductance and *R *[Ω] the resistance of a resonance circuit. Thus the overall power loss (Eq. 1) is reduced by the factor *R*_ov_/(*R*_ov_+*R*_hs_). Only the part *R*_hs_/(*R*_ov_+ *R*_hs_) of this reduced total power loss occurs at the break. It is given with respect to the total power loss of the intact resonator *P*_loss _by



and has the maximum value with respect to *P*_loss _of Eq. 1 at *R*_hs _= *R*_ov _yielding [11]



#### Analytical solution for a hot spot

An analytical description of the thermal uptake around a hot spot with respect to the metallic structure, the electrical paths of the resonance circuit and different power loss mechanisms is not possible. An easy analytical description is possible for a sphere with radius r_sphere _emitting a constant power *P *uniformly from the sphere surface inside a homogeneous medium surrounding the power emitting sphere disregarding blood perfusion. This model is a good approximation for a point-like power source. After reaching the thermal equilibrium the constant power penetrates through every spherical surface surrounding the power source in the sphere center independent of the radius *r *(with side condition *r *> r_sphere_). The temperature difference Δ*T *[K] between a point at the hot spot surface and a point far away (∞) from the power source, which for a living system is a point with the normal body temperature, can be calculated in a homogeneous medium for a power loss *P *from the equation for heat diffusion,





where λ[W m^-1 ^K^-1^] is thermal conductivity. Equation 3b can be resolved for the critical radius r_crit_, which describes the distance below that a critical temperature increase Δ*T*_crit _is exceeded. From the critical radius r_crit _the critical volume of the sphere with temperature increases above Δ*T*_crit _can be calculated.

### Worst case parameters for a hot spot on a linear wire

The worst case scenario is described by the following conditions.

1. For a maximum power loss the axis of the resonator has to be perpendicular to the main magnetic field. For a circularly polarized transmit coil this statement is sufficient, whereas for a linearly polarized transmit coil in addition the axes of the resonator and the transmit coil have to be parallel.

2. The resonator is exposed to an MR sequence with the maximum allowed rf power corresponding to a maximum SAR (4 W/kg).

3. The quality factor of a resonator embedded in tissue has the maximum value achievable with the volume of the resonators inductance (experimental data are taken from [11]). For a resonator with a given volume this condition implies, that the product *V*_ind _*Q *has a maximum value.

4. The resistance R_hs _at the rupture of the circuit path is equal to the overall resistance R_ov _of the formally intact resonator. Then Eq. 2b applies.

5. The resonator is exposed to the MR sequence over a long time. Therefore the thermal equilibrium is approached.

6. The tissue near the hot spot is not cooled by blood perfusion [17].

7. The tissue near the hot spot is not cooled by blood flow.

8. A temperature increase of 5 K is already assumed as critical, because it is the starting point for induction of first irreversible cell destructions [18-22].

Beside the above defined worst case scenario, which is carefully analyzed, also conditions of lower risk are of interest. The effect of weakening the topics 1–4 can be simulated by reducing the power loss of the hot spot. The most important and unpredictable parameter is the resistance R_hs _of the rupture of the circuit path (topic 4). Of course, it is not very probable that R_hs _is close to R_ov _but it may happen. All worst case assumptions as well as the physical parameter of the wire are varied to check the influence on the results. If the risk is also existent under near worst case conditions or, more dangerous, under conditions not close to the worst case, MRI active devices as implants for overcoming the Faraday cage effect are definitely not safe.

### Finite volume simulation

Because the analytical solution of Eq. 3 overestimates the real situation by neglecting the metal of the implant with the capability of a faster thermal distribution of the applied energy, a finite volume simulation is used, which can also take into account blood perfusion and blood flow. This finite volume simulation uses the bioheat transfer equation.

During the last fifty years since Pennes publication [15,16] on the bioheat transfer equation, there have been many attempts to solve this equation more accurately for biological tissue. This problem is not completely solved with respect to all possible physical parameters. The influences on the heat transfer in biological tissue are the thermal conductivity λ of tissue, the heat transport due to blood perfusion w_b _([(m^3^) of blood × (m^-3^) of tissue × (s^-1^)], sometimes also cited as [(kg) of blood × (m^-3^) of tissue × (s^-1^)]), the heat transport due to blood flow in larger vessels, the metabolic heat production *Q*_met _inside living tissue and the applied power *P*_ex _to the body from external sources. This investigation is a test on persisting dangerous conditions using near worst case assumptions and therefore uses simplifications. For the comparison with the analytical model only the heat transport by the thermal conductivity of tissue is considered. Other calculations also take into account the influence of the metallic wire as well as blood perfusion. Some simulations additionally estimate the volume with a critical temperature increase around a hot spot inside a vessel wall with a cooling blood flow inside the vessel lumen in a very short distance from the hot spot.

The simulations are not performed with commercially available software. The algorithm is self-coded for problems with cylindrical geometry in Kylix and Delphi, a software development environment, based on object oriented Pascal. The graphical outputs are mostly generated by an evaluation version of Teechart 7 (registered β-test) used within the Delphi and Kylix environment. The implemented algorithm is essentially the same as the one in the first part of the investigation [12], but it is generalized to cover also blood perfusion. As physically realistic and properly working devices hardly reach critical temperature increases, it was not necessary to incorporate blood perfusion to the algorithm used in the first part of the investigation [12]. On the other hand hot spots easily reach critical temperature increases and therefore it is necessary to look at blood perfusion and blood flow, which may make the results less critical.

The *total simulation *volume is a cylinder with length 2L_sim _and diameter 2R_sim_. This is adequate for a straight wire along the cylinder axis with radius r_wire_. The chosen geometry allows the use of cylinder coordinates (*r*, *φ*, *x*) and reduces the calculation time taking advantage of two symmetries. Firstly, the model needs not to consider *φ *in cylindrical coordinates because of the cylindrical symmetry and can use finite volumes only dependent on *r *and *x*.

**Figure 1:**
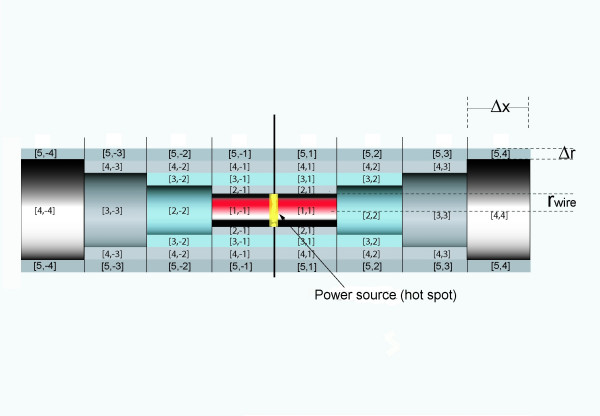
**Total simulation volume with hot spot at the symmetry plane and cell definitions**. Total simulation volume for calculations with the power source generating the hot spot at position x = 0 (yellow depicted) and convention for cell labeling. The calculation volume is only half of the total simulation volume and is defined by positive indices.

**Figure 2:**
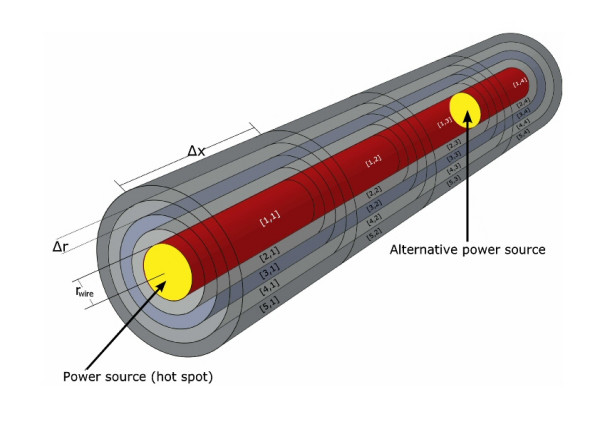
**Calculation volume with alternative hot spot locations and cell definitions**. Calculation volume with power source generating the hot spot between two cell elements (yellow depicted). The image shows an example of a small calculation volume and the convention for cell labeling of the algorithm. The power source for most cases is assumed to lie within the symmetry plane at x = 0. Half of the total applied power reaches the cell with index i = 1 and j = 1 for r and x respectively. For simulating a planar heat sink the power source is shifted to a different position between cell elements at position x_f-1 _and x_f _(also yellow depicted). In that case half of the entire applied power is assumed to reach the cells at position x_f-1 _and x_f_.

Secondly, a plane of mirror symmetry exists, which is orthogonal to the *x*-axis of the cylindrical coordinate system and divides the total simulation volume into two parts (Figure 1). The simulation needs only to calculate the temperature map at one side of this mirror plane, because the temperature values on the other side are identical (Δ*T *[*r*,-*x*] = Δ*T *[*r*,*x*]). The *calculation volume *with length L_sim _and radius R_sim _(Figure 2) therefore is only half of the total simulation volume with length 2L_sim _and radius R_sim_. The simulation calculates maps of temperature increases developing in time around a hot spot for a constant hot spot power loss *P*_hs_. The calculation volume is divided into many small finite volumes, also called simulation cells. Utilizing the symmetries the cylindrical calculation volume with length L_sim _and radius R_sim _is divided into the following cells C[i,j]. It consists of n sub-cylinders with length Δ*x *and with center-positions x_j _= (j-1/2)Δ × (j = 1, 2, ...., n) on the cylinder axis. Each sub-cylinder of length Δ × is divided in one inner cylinder with radius *r*_wire _(i = 1) and m-1 shells of thickness Δr.

The energy exchange Δ*E *[J] between two simulation cells with a specific contact area A[m^2^], a temperature difference Δ*T *[K] and a heat diffusion path length d[m] during a time interval Δ*t *[s] is given by the equation for heat conduction as



For the following, the sign of Δ*E *for a cell C[i,j] has to be chosen such, that it is positive for receiving energy and negative for an outgoing energy. The total energy change Δ*E*_tot _of one cell during a time interval Δ*t *is the sum of all exchanges with adjacent cells with non-zero contact area and the energy change due to a heating power *p*_cell _inside the cell.



*p*_cell _is non-zero only at the hot spot, i. e. in the two cells at the assumed two fracture surfaces of the wire. The temperature increase ΔT*[K] of one cell during one iteration step can be calculated by



where c[J/(kg K)] is the specific thermal capacity of the cell material, *V*_cell _[m^3^] is the cell volume and ρ[kg/m^3^] is the density of the material of *V*_cell_.

Each cell C[i,j] (2 < = i < = m, 1 < = j < = n), except those at i = 1 (radial direction) has contact to 4 adjacent cells with contact areas different from zero (Eqs. 7a-c). These contact areas as well as the volumes of the cells (Eq. 7d) only vary with the index i.



A_x_(i) = π·⌊(r_wire _+ (i - 1)·Δr)^2 ^- (r_wire _+ (i - 2)·Δr)^2^⌋     i = 2, 3, 4,....,m      (7b)

A_r_(i) = 2·π·(r_wire _+ (i - 1)·Δr)·Δx     i = 1, 2, 3,...,m      (7c)

V_cell_(i) = A_x_[i]·Δx     i = 1, 2, 3,...,m      (7d)

A_x_(i) is the contact area in both directions of the cylinder axis (from any index j to j-1 and to j+1) whereas A_r_(i) is the contact area in radial direction from index i to i+1. The contact area in radial direction from index i to i-1 is identical to the area A_*r*_(i-1). At i = n and j = m the calculation volume has boundaries. These boundaries are implemented as boundary cells at index m+1 (r-direction) and n+1 (x-direction), which work as an ideal heat sink. The boundary condition for this heat sink is dT/dt = 0, which keeps the temperature of our boundary volume constant (Δ*T *= 0), even when receiving energy during one simulation step with duration Δ*t*. The condition Δ*T *= 0 at the outermost cells can describe on one hand, the behavior of the human body to keep its temperature nearly constant by regulating the energy transport. In our model this temperature regulation allows to shift the condition Δ*T *= 0 closer to the hot spot. On the other hand Δ*T *= 0 can also be a model for rapid heat transport through flow inside a larger vessel near the hot spot. A fast blood flow, which transports immediately all applied energy to the entire blood pool with an infinite thermal capacity, can be simulated by a situation, where the hot spot is close to boundary cells, which keep the temperature constant even if receiving energy. At i = 1 as well as j = 1 the calculation volume has boundaries without energy exchange. For index i = 1 no cells with lower index i (radial direction) exist and therefore no energy exchange is possible. For index j = 1 with cell center position Δx/2 the symmetry plane defines an identical temperature at – Δx/2 (Figure 1), with no energy exchange across the symmetry plane. For simulations with the hot spot on the symmetry plane only one cell C[1,1] receives power during the simulation process, which is half of the total hot spot power. The corresponding half power is applied to the part at the opposite side of the symmetry plane, which is unnecessary to calculate. For simulating the influence of blood flow inside a vessel close to a hot spot, the hot spot is placed near the heat sink (at n+1) between cell elements C[1,f-1]; C[1,f], where f is close to n. Subsequently half of the total power is applied to each of the corresponding cell volumes. For this case the symmetry plane of the simulation results in two symmetrically placed hot spots, which are situated in the total volume at both sides of the symmetry plane. If the distance between the symmetry plane and the hot spot is large enough, the two hot spots do not influence each other. Instead of choosing a very large distance between the hot spot and the symmetry plane, which would require many cell elements with a resulting long calculation time, alternatively an additional circular shaped planar heat sink can be implemented at the symmetry plane to suppress the influence of the non existing second hot spot.

The applied energy Δ*E *= *p*_cell_Δ*t *heats up only the corresponding cell volumes (C[1,1] or C[1,f], C[1,f-1]) during one iteration step with duration Δ*t*. The distribution from this (these) power receiving cell(s) to adjacent cell elements is only due to heat conduction on the wire and through tissue during an iteration step. Additionally part of the energy of each cell can be transported out of the total simulation volume by blood perfusion. The calculation volume consists of a two dimensional field of cells C[i,j] (i = 1, 2, ...., m+1; j = 1, 2, ...., n+1).

All metal wire cell elements have the index i = 1. They all have identical freely definable physical parameters λ, ρ and c. The parameters for all other cell elements are set to the values of tissue (table 1). The different thermal conductivities of metal and tissue require a changed diffusion path length at the metal-tissue interface. Assuming the heat conduction takes place from the center of a cell (arithmetic mean of inner and outer cell limit with respect to *x *and *r*) to the center of the adjacent cell, the heat conduction for such an interface takes place over two different materials with only part of the diffusion length for each of the materials (r_wire_/2 for metal and Δr/2 for tissue). Because the thermal conductivity of tissue is much lower compared to that of metal (table 1) a sufficient approximation is the use of only the diffusion length through tissue. For part of the simulations, in which the metallic wire is replaced by tissue, the heat diffusion length at the cylinder axis is (r_wire_+Δr)/2.

**Table 1:** Physical constants of tissue, titanium, iron and tantalum

material	density ρ	specific heat c	thermal conduct. λ
	[kg/m^3^]	[J/(kg K]	[W/(m * K)]
tissue	1000 [22]	3650 [23]	0.5 [24]
titanium	4510	523	21.9
iron	7870	449	80.2
tantalum	16680	140	57.5
niobium	8580	265	53.7

The entire simulated time *t*_sim _consists of q iterations with time interval Δ*t *(t_sim _= q Δ*t*). The simulation starts at *t *= 0 with a temperature field Δ*T*[i,j] = 0 for all indices i and j respectively. For each iteration step with duration Δ*t *the total energy change Δ*E*_tot _[i,j] for each cell *C*[i,j] is calculated according to Eqs. 4, 5 respecting the related contact areas (Eqs. 7a-7c) and the corresponding diffusion lengths. From Δ*E*_tot _[i,j] the temperature increase ΔT*[i,j] is calculated according to Eq. 6 respecting Eq. 7d. This value is added to the prior value according to

Δ*T*_new _[i,j ] = Δ*T*_old _[i,j ] + ΔT*[i,j ] – w_b _· Δ*T*_old _[i,j] · Δ*t *      (8)

for each cell during the whole iteration process. Eq. 8 respects a perfusion term w_b_. It describes which part per second of a tissue volume is exchanged by perfusion against new blood from the arterial blood pool. The new part has to be heated from Δ*T *= 0 to the increased temperature level and therefore the temperature increase of the cell volume C[i,j] is reduced. The implementation of Eq. 8 is similar to the use of Pennes equation. In contrast to Pennes equation, Eq. 8 neither assumes different physical parameters of blood and tissue, which would lead to an additional factor modulating w_b_, nor uses a temperature dependent blood perfusion parameter, which increases with enlarged temperatures. It is sufficient to perform a simulation with an overall increased constant blood perfusion to check the persistence of physiological critical circumstances. Furthermore no metabolic heat production is assumed. The calculated Δ*T*[i,j] is assigned to the arithmetic mean of inner and outer cell limits with respect to *x *and *r*, respectively.

### Control of simulation results

The implemented algorithm was controlled with different checks. Firstly, the numerical simulation for a tissue only environment is compared to the analytical model. Secondly, for each numerical simulation the totally applied energy at the end of the simulation (*W*_total _= *Pt*_sim_) is compared to the energy stored inside the simulation volume, computed from the final temperature increases of each cell respecting the heat capacity, added with the energy, that has left the simulation volume to any heat sink and the energy needed during the total simulation process for heating up blood from the arterial blood pool due to the perfusion term of Eq. 8. Thirdly, the algorithm was tested, whether it provides similar results for identical geometries with different spatial or different temporal resolution as well as for different sizes of the total simulation volume surrounding the hot spot.

### Analytical solution for thermal equilibrium as worst case of a hot spot embedded in homogeneous tissue

The variable parameter for the analytical model according to Eq. 3b is the hot spot power *P*_hs_. The hot spot power depends on the volume and the quality factor of the resonator (Eqs. 1, 2b), but as well on the orientation of the resonator with respect to the main magnetic field and the SAR of the applied MRI sequence. Prior experimental experiences [11] reveal quality factors below 4 for reasonably constructed resonators embedded in 0.9 % physiologic saline solution as a model for tissue. As worst case in this investigation a quality factor of 5 is assumed. With a known quality factor *Q*, the resonator volume and the knowledge of the applied MRI sequence the maximum hot spot power P_hs _can be calculated according to Eqs. 1 and 2. Alternatively, Eqs. 1 and 2 can be used to calculate the smallest inductance volume of a resonator, which effects – using worst case conditions for the quality factor (Q = 5) and MRI sequence (const = 4 mW/cm^3^) – a certain critical hot spot power loss.

### Simulations

Temperature maps were calculated depending on different physical parameters respecting partly blood perfusion and blood flow inside a vessel near the hot spot. Unless otherwise noted, the following standard parameters were used for the simulation: titanium wire with 50μm radius, hot spot power of 100 mW, normal perfusion rate w_b _= 0.00125 m^3 ^m^-3 ^s^-1 ^[17], simulation time of 900 s (according to FDA regulations 900 s are the maximum tolerable exposure time of the trunk for MRI sequences with a specific absorption rate of 4 W/kg). The critical temperature increase for calculating the critical volume is set to 5 K corresponding to a tissue temperature of 42°C. The simulations investigate the following eight topics.

1. Spatial and temporal resolution: The metal tissue interface is a critical part of the simulation. The simulations with varying spatial resolution in both directions as well as a better temporal resolution allow the assessment of the temperature differences at the metal tissue interface.

2. Time development:

a. Temperature maps for a tissue-only simulation: The pure tissue temperature maps were calculated with normal blood perfusion (movie 1 in Additional file 1), whereas the calculations for the comparison with the analytical solution for thermal equilibrium (Eq. 3b) were calculated without taking into account blood perfusion.

b. Temperature maps for a titanium wire: The temperature maps were calculated without and with normal blood perfusion to indicate the changes (movie 2 in Additional file 2).

**Figure 3:**
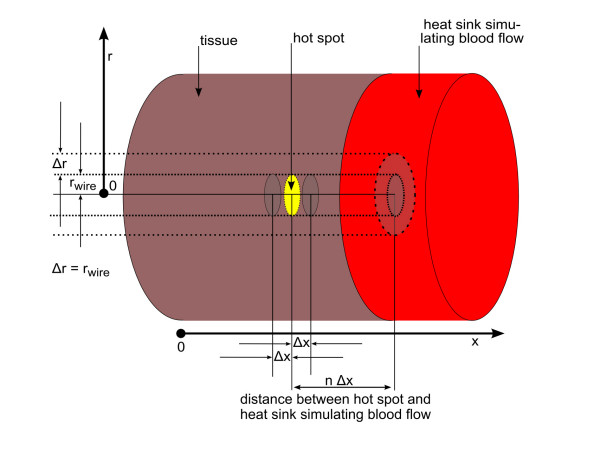
**Definition of the heat sink used for the simulation of blood flow inside a vessel close to the hot spot**. Schematic of a hot spot embedded in tissue with a circular planar heat sink simulating a close blood flow inside a larger vessel. The calculation volume only consists of tissue, because a metallic wire has to have a radial direction, which is not possible with the chosen cylindrical geometry. Half of the total applied power reaches each cell element on the right and left to the indicated hot spot (yellow). All energy reaching the heat sink is immediately transported away, which is implemented as cell elements never changing the temperature (Δ*T *= 0) even if receiving energy.

3. Hot spot power: The final temperature maps were calculated with varying hot spot power loss *P*_hs_. The variation of the hot spot power *P*_hs _corresponds to a change of implants, because their total power loss depends on the inductance volume of the implant (*V*_ind_) and the quality factor *Q *of the resonance circuit. A larger hot spot power is equivalent to a better quality factor and/or a larger inductance volume. Also a decreased hot spot power can simulate a resonator not perfectly aligned perpendicular to the main magnetic field or an MRI sequence without maximum SAR.

4. Material: The final temperature maps were calculated for four different metals of the linear wire (titanium, niobium, tantalum and iron) to test influences of the thermal conductivity.

5. Radius: The final temperature maps were calculated with varying radius of a titanium wire (30 μm – 500 μm) to check the influence of the increased heat transport capability of a metallic wire with a larger radius.

6. Perfusion rate: The final temperature maps were calculated with normal and increased perfusion rates (from 0.00125 m^3 ^m^-3 ^s^-1 ^to 0.02 m^3 ^m^-3 ^s^-1^) for tissue.

7. Blood flow: Blood flow is modeled as a heat sink, that is very close to the hot spot, and immediately transports all applied energy out of the simulation volume. This model can be integrated into the finite volume simulation by reducing the distance between the hot spot and the implemented heat sinks, which are at the boundaries of the simulation volume representing a circular cylinder. Either the lateral cylinder surface is moved closer to the hot spot by reducing the cylinder radius, or the hot spot is moved close to the circular cylinder top at position x_n+1_. Unfortunately, the first model with the wire on the cylinder axis and a heat sink surrounding the hot spot in close distance does not represent a typical anatomical condition: a hot spot inside a vessel wall is not surrounded in this way by blood flow. Therefore, this model is not considered further. A better model is a planar blood flow in proximity (right side of the hot spot in Figure 3). Consequently, the hot spot on the axis of the cylindrical volume is placed near the cylinder top representing a planar heat sink. In detail, the hot spot is placed between the cell elements at position x_f-1 _and x_f_, where the index f is close to the maximum index n. Because the hot spot in this case is placed outside the symmetry plane, the symmetry plane automatically defines the existence of a second hot spot. To avoid any influence of the second unwanted hot spot, a sufficiently large distance between them is chosen or a third heat sink is implemented at the position of the symmetry plane to suppress the influence of the second hot spot. In the model of the planar blood flow, the metal wire on the cylinder axis is orthogonal to the planar heat sink. This is not the realistic situation, where the wire should be parallel to the planar heat sink. Therefore, those calculations are done without a wire and all cell elements, also those on the cylinder axis, are assumed to be tissue.

8. The critical temperature for the calculation of the critical volume is varied from 5 K with first assumed cell destructions to 20 K, which should be near to a total cell damage even for very short exposure times.

## Results

### Analytical solution for thermal equilibrium

**Figure 4:**
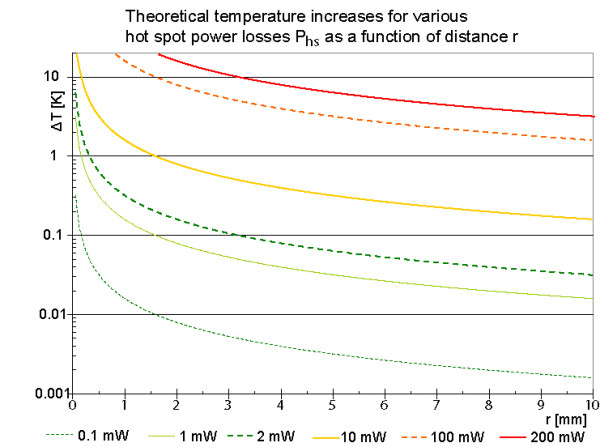
**Theoretical final temperature increases around a spherical power source for various power losses**. All five curves start at a radial distance r = 50 μm from the hot spot center, because 50 μm is the typical wire radius. It is assumed that the hot spot is surrounded by tissue only. For power losses of 2 mW or more the temperature increases exceed 5 K. Because of the cubic dependence of the critical volume on the critical radius the critical volume is substantial for power losses above 10 mW.

The analytical model describes the worst case in all aspects, because it assumes an indefinite exposure time to the excitations of an MRI sequence without respecting any blood perfusion (w_b _= 0 m^3 ^m^-3 ^s^-1^) or blood flow. Figure 4 shows the temperature curves according to Eq. 3b for different power losses and distances up to 15 mm. Because of the cubic dependence to the critical radius r_crit _the critical volume V_crit _can be easy calculated.

**Figure 5:**
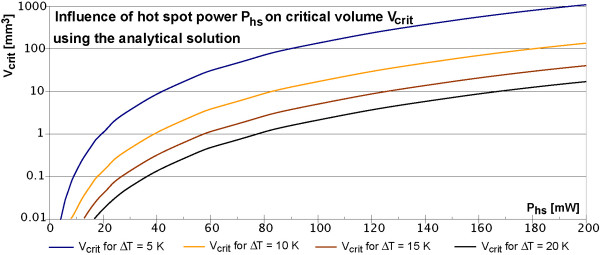
**Critical volume in dependence of hot spot power P_hs _for various critical temperatures**. Using the analytical solution for thermal equilibrium of equation 3b a spherical critical volume can be calculated using a critical temperature. The images show examples for 4 different critical temperature increases up to 20 K. For a hot spot power of 200 mW a volume of about 20 mm^3 ^reaches temperature increases of more than 20 K.

The analytical solution for *r *= 0 is infinite, but distances below 50 μm are irrelevant, because the radius of stent struts, which defines the size of a hot spot, has a similar dimension. The red horizontal double line at 5K indicates an assumed physiologically critical temperature increase with first irreversible cell destructions, which is taken from the literature on hyperthermia [18-22]. At 2mW hot spot power this low value of 5 K is just exceeded for r < 64 μm. This value is so close to the hot spot size, that 2 mW may be considered as save. The analytical solution as worst case scenario is further considered in Figure 5. It shows for 4 exemplary critical temperature increases (5 K, 10 K, 15 K and 20 K) the critical volume V_crit _in dependence on the hot spot power.

**Figure 6:**
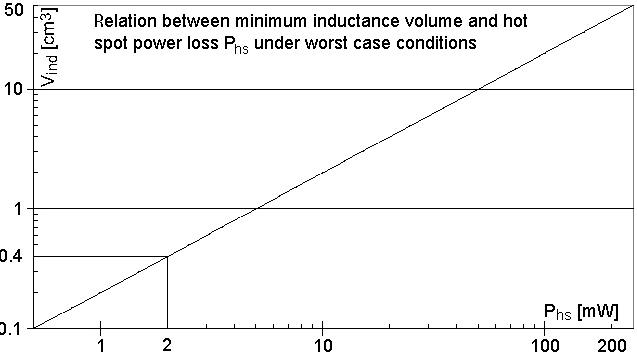
**Maximum hot spot power P_hs _as a function of inductance volume V_ind_**. The maximum hot spot power for a given inductance volume is calculated using equation 1 and 2b under worst case conditions (Q = 5, const = 4 mW/cm^3^). It is a linear relation in log-log presentation. Inductance volumes below 0.4 cm^3 ^are definitely safe.

A certain hot spot power can be converted into the smallest inductance volume capable of setting up this hot spot power *P*_hs_. Considering again the worst case scenario (combining Eqs. 1 and 2b) a linear relation between the hot spot power P_hs _and the volume of the implants inductance *V*_ind _exists, where the constant of proportionality is determined by the maximum quality factor *Q*_max _of a resonator inside tissue and the maximum power loss density P_V _of table 2. With the experimentally determined value of *Q*_max _= 5 [11] the linear relation between *P*_hs _and *V*_ind _is that of Figure 6; for convenience both axes are scaled logarithmically.

**Table 2:** Data for calculation of the power density *P*_V _of an active implant referring to Eq. 1 for an MRI sequence with an SAR of 4 W/kg (manufacturer declaration)

permeability of vacuum	μ_0_	[V s/(A m)]	12.6E-7
magnitude of magnetic excitation field	B_1_	[μT]	25
repetition time of MRI sequence	TR	[s]	2.23
duration of one excitation	τ	[ms]	0.80
number of identical excitations during TR	N		246
duty cycle c_dc _N τ/TR	c_dc_		0.09
pulse waveform modulation factor c_pwm_	c_pwm_		0.45
Larmor frequency = resonance frequency of LC circuit	ν_0_	[MHz]	63.8
ω_0 _= 2π ν_0_	ω_0_	rad/s	4.0 10^8^
power loss density P_V _= P/(Q V_ind_) (Eq. 1)	P_V_	[mW/(cm^3^Q)]	4.0

Looking at the graph in Figure 6 only volumes larger than 0.4cm^3 ^can develop a hot spot power above 2 mW, which is, according to Figure 5, the minimum power for temperature increases above 5 K. Stents for coronary arteries have a maximum dimension of 5 mm in diameter and 20 mm in length. So their volume is below 0.4 cm^3^; this means they are safe even under worst case conditions.

### Comparison of the analytical solution with the finite volume simulation

**Figure 7:**
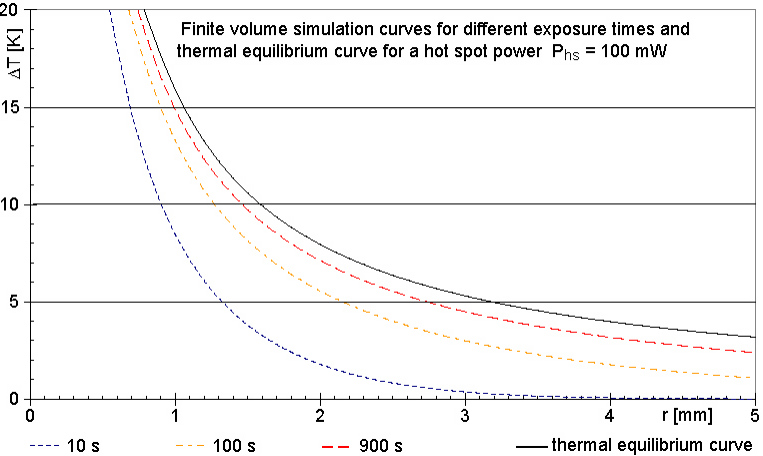
**Calculated temperature increases around a spherical power source of P_hs _= 100 mW after different simulated times**. The four graphs of the calculation (25 μm in r-direction, 125 μm in x-direction, temporal resolution 200 μs) approach the theoretical curve (black line) of final temperature increases according to thermal equilibrium, as expected. This is one indication of a correctly implemented algorithm. All curves refer to a power loss of 100 mW. The black line of figure 7 and the 100 mW line of figure 4 are differently scaled presentations of the same case.

Figure 7 shows a comparison of the analytical solution for thermal equilibrium with the simulations after different simulated times (see also movie 1 in Additional file 1). For this case the physical parameters of the wire with radius r_wire _= 50 μm are set to those of tissue and the parameter of blood perfusion w_b _is set to zero (Eq. 8). Even though the cylindrical geometry is not the best choice for a spherical problem, the simulation results should converge to the analytical solution, if the simulation time is sufficiently long and the simulation volume is sufficiently large. Figure 7 shows the calculated curves in radial direction for different times as well as the analytical solution for identical parameters. With increasing simulated time the calculated curves approach the thermal equilibrium curve verifying a correct implementation of the algorithm.

### Temporal and spatial resolution

As long as the temporal resolution is high enough to prevent the simulation results from an oscillatory behavior, an increased temporal resolution does not change the calculated maps. Therefore it is sufficient to use the lowest possible temporal resolution for a certain spatial resolution.

**Figure 8:**
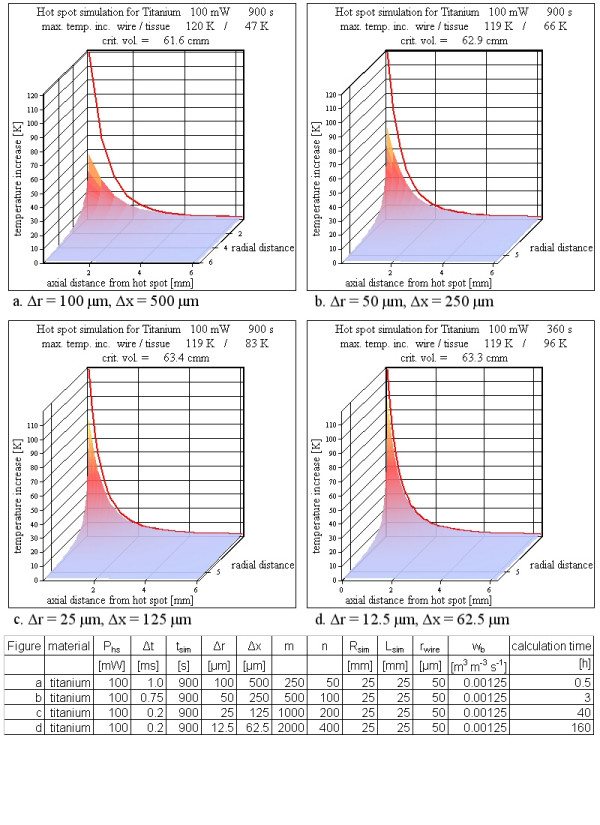
**Various spatial resolutions**. The spatial resolution influences the simulation results. With better spatial resolution the tissue temperature increases close to the wire approach the nearly unchanged red line representing the wire temperature increases. However, the calculated critical volumes are hardly affected by the spatial resolution. A better spatial resolution requires a better temporal resolution to obtain correct results. As a consequence, the calculation time increases from 3 min to 160 h using a PC with a 3 GHz processor (see table below figure).

An increased spatial resolution as well as an increased thermal conductivity of the wire always requires an increased temporal resolution to exclude erroneous results. With better spatial resolution in radial direction (keeping the wire diameter constant) the wire temperature curve and the tissue temperature directly adjacent to the wire approach to each other with a predominant increase of the tissue temperature. A better spatial resolution just in axial direction changes mainly the maximum temperature increase on the wire. This is obvious, because with a shorter cell volume in axial direction, the center of the cell moves closer to the hot spot, where the analytical model has an infinite temperature increase. Changing both spatial resolutions by the same factor combines the two effects; it leaves the temperature increase on the wire nearly unchanged and reduces the differences between wire and tissue temperatures (figure 8). For all three cases the changes of the critical volume using a definite temperature increase stays within ± 3 %. Figure 8 shows the temperature map of tissue as a surface in a 3D perspective view and the temperature of the wire with radius 50 μm as a red line at r = 0 mm. With increased spatial resolution in both directions, the temperature increases on the wire stay nearly constant (compare Figures 8a, 8b and 8c) and the temperature increases of the tissue directly adjacent to the wire get closer to the wire curve. Because the wire temperature curve seems not to change between the Figure 8a, 8b and 8c, this behavior is tested with an even better spatial resolution (Figure 8d). No significant changes of the temperature increases on the wire occur, but the adjacent tissue temperature increases get closer to the wire values, as expected. Because of the unacceptably long calculation time of about a week and the theoretical possibility to correct the values of tissue close to the wire by an interpolation using the almost unchanged wire temperature curve, this increased spatial resolution was tested only once and is not applied to other simulations. For the shown examples in Figure 8 the resolution in radial direction is superior to that in axial direction to keep the temperature differences between wire and adjacent tissue small.

### Time development

#### Time development for a tissue only simulation

Movie 1 (see Additional file 1) presents the time development for a hot spot (100 mW) completely embedded in tissue with the physical tissue parameters from table 1 and with normal blood perfusion. Figure a of movie 1 shows the calculation volume. Already after 5 ms the temperature increases directly adjacent to the hot spot reach high values. Because of this large peak near the location of the hot spot, the rest of the information is hidden. This hidden information is shown in Figure b, which is identical to Figure a, apart from the fact that temperature increases larger than 10 K are truncated to 10 K. This allows the visualization of the temperature map around the physiologically critical temperature of the hot spot more clearly. Figure c uses the known symmetries to show a cut along the cylinder axis with the failure at the center of the total simulation volume. Again, temperature increases larger than 10 K are truncated to 10 K. Figure d is identical to Figure c but instead of a perspective surface 3D view, a map including the wire with the temperature increases coded in colors is shown. This helps to identify the 'area' inside the r-x-plot where the starting point of physiologically critical temperature increases of 5 K [18-22] is exceeded. Figure d also shows the critical distance in *r*- and *x*-direction respectively, as well as the critical volume with temperature increases above 5 K. At the end of the movie, two views are shown alternately at a simulated time of 900 s. Both views are identical in all simulation parameters except the simulation volume. The size of the simulation volume is doubled in *x *direction as well as in *r *direction. This shifts the energy (heat) sink further away from the hot spot itself. One of the alternating results was calculated using a 250 × 250 matrix for a size up to 12.5 mm for *r *and *x *respectively. The second map was calculated for a 500 × 500 matrix for a size up to 25 mm for *r *and *x *respectively. Only the inner 250 × 250 points are plotted for the comparison of both calculations. It can be seen that the temperature distribution is almost identical apart from the fact, that for *x *≈ 12.5 mm and *r *≈ 12.5 mm the simulation with more cells shows a slight deviation from the zero line. The simulation with the smaller matrix shows a straight zero line at r_250 _and x_250_, which is obvious, because this is the boundary condition for this simulation. The small difference between both simulations points out, that the boundary condition with a heat sink works very well as long as the absolute value of the gradient at the boundary is low. The effect of the heat sinks can also be seen in the accumulated energy that has left the simulation volume to any heat sink during the simulated time (at n+1, at m+1 and due to blood perfusion). For the smaller simulation volume 71 J of the applied total energy of 90 J leave the simulation volume. For the larger volume the value is reduced to 45 J. Without blood perfusion the 'lost' energy for both volumes decreases to 67 J and 20 J respectively. The critical volume with temperature increases over 5 K is only moderately reduced by normal blood perfusion. Without blood perfusion it is 72 mm^3 ^and 86 mm^3 ^for the small and large volume respectively, whereas the critical volume with blood perfusion reaches 59 mm^3 ^and 64 mm^3 ^(see Additional file 1).

#### Time development for a titanium wire

As comparison to the tissue-only simulation the second movie (see Additional file 2) presents a hot spot (100 mW) between the rupture surfaces of a titanium wire with radius 50 μm. The results are similar to the tissue-only simulation. In figure a and b, a perspective surface 3D-view shows the temperature map for the tissue part of the simulated volume. The temperature curve for the metal wire is shown as additional red line inside figure a and b. In figure c, only the map for tissue is shown for the total simulation volume, whereas in d the temperature of the titanium wire as well as the tissue temperature is coded in colors. At the end of the movie alternately the simulation after 900 s with and without normal blood perfusion is shown. It can be seen that the critical volume is reduced by blood perfusion (from 85 mm^3 ^to 63 mm^3^).

### Hot spot power

The variation of the hot spot power corresponds – for a specific MRI sequence (see table 2) – to a variation of the product between the resonator volume and the quality factor of the implanted resonating circuit, to which the hot spot power is proportional (Eq. 1). Assuming a constant volume, *P*_hs _is linearly dependent on the quality factor *Q*, whereas assuming a constant *Q*, *P*_hs _is linearly dependent on the volume.

**Figure 9:**
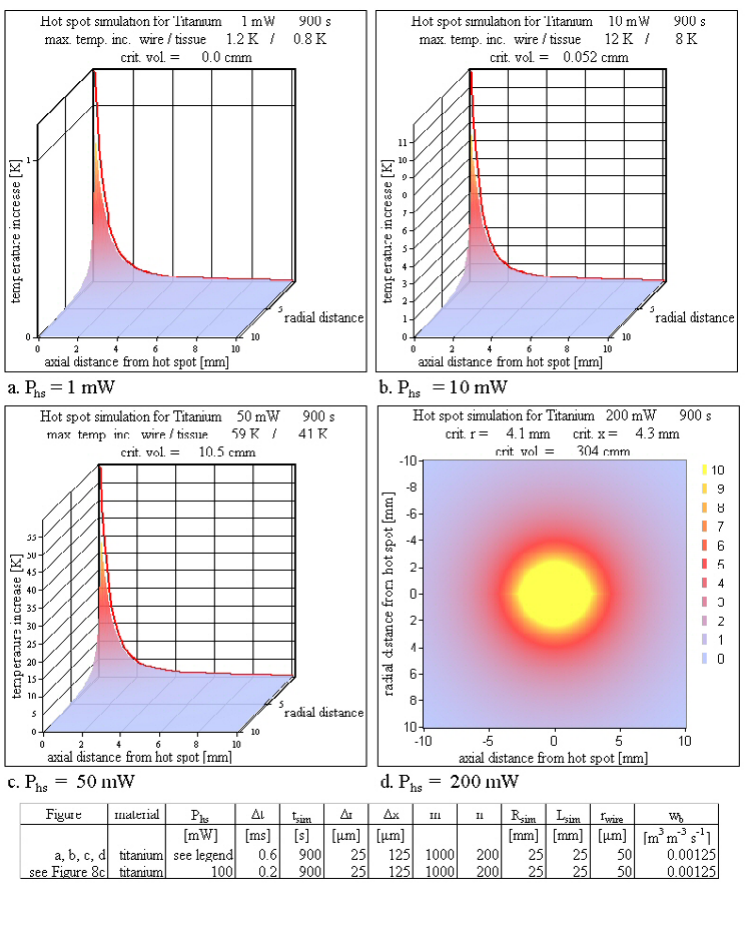
**Various hot spot powers P_hs_**. Varying the hot spot power leads to an increasing critical volume up to more than 300 mm^3^. For power losses greater than 100 mW the tissue temperature exceeds the boiling point of water. This high temperature is reached within a few ms (see Additional file 1 and Additional file 2).

The step from the worst case (infinite exposure time to the rf pulses of the MR-system and pure tissue model) to the near worst case situation with an exposure time of only 900 s and with a model of a metallic wire in tissue shifts the lowest hot spot power loss *P*_hs _to reach a critical temperature increase above 5 K inside tissue from 2 mW to 10 mW (Figure 9b). For 100 mW (Figure 8) and 200 mW (Figure 9d) the simulations show a large critical volume with temperature increases above 5 K. This is best pictured in Figure 9d, which shows a slice through the hot spot along the symmetry axis (the wire) at *r *= 0 m. The critical distances from the hot spot to the location, where the temperature increase equals the critical value of 5 K, reach a few mm in *r *and *x *direction respectively, and the total tissue volume with temperature increases over 5 K reaches about 300 mm^3^.

### Material

**Figure 10:**
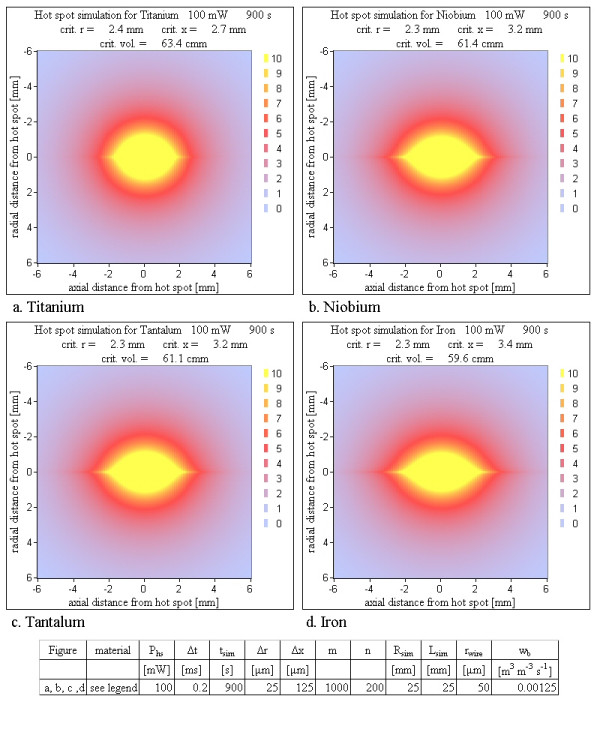
**Various wire materials**. A hot spot in pure tissue without wire material would yield a temperature distribution of spherical symmetry. A wire with its high thermal conductivity improves the heat distribution in axial direction; the sphere is stretched in axial direction and compressed in radial direction. This effect is weakest for titanium and strongest for iron (see table 1). With increasing thermal conductivity the critical volume becomes slightly smaller (only 7 % change). The simulations show, that the different thermal conductivities of the metals are not an important factor concerning the critical volume.

Because of the better thermal conductivity of a metallic wire compared to tissue, the introduction of the metallic wire, which is present for all real cases, lowers the risk of the analytical situation with the assumption of a hot spot inside homogeneous tissue. Various wire materials distribute the heat more or less efficiently in wire direction depending on the thermal conductivity. For example, iron has a thermal conductivity about four times as large as that of titanium (table 1). However the differences in the simulation results between the analyzed metals are small. With increasing thermal conductivity the critical volume is slightly reduced and the final shape is stretched in axial and compressed in radial direction (Figure 10).

### Radius

**Figure 11:**
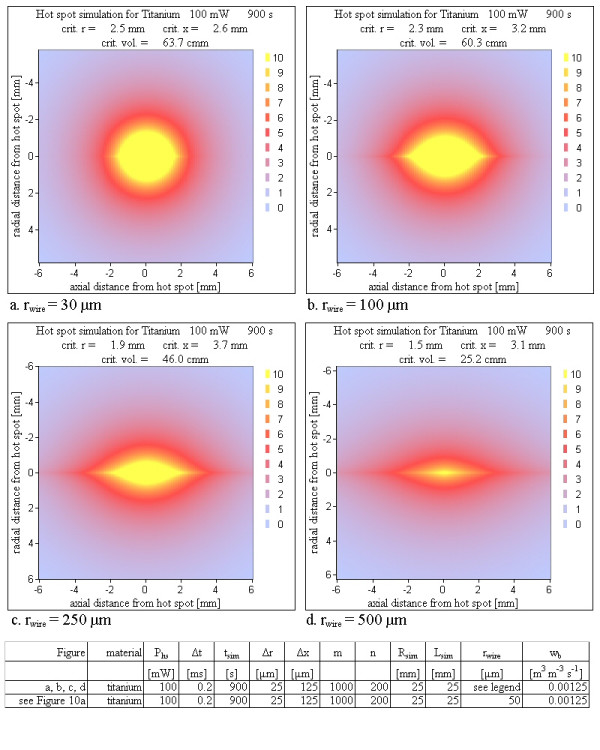
**Various wire radii**. An increasing wire radius with a larger capability of heat transport along the wire improves the heat distribution in axial direction with the same consequences as in figure 8. With increasing wire radius the critical volume becomes smaller, but it stays unacceptably large even for an unrealistic wire radius of 0.5 mm (1 mm diameter).

As expected, the heat is distributed more efficiently with increasing radius and therefore the critical volume drops, but even for a titanium wire with radius of 0.5 mm (1 mm diameter), a hot spot power of 100 mW causes a critical volume of 25 mm^3 ^(Figure 11). The more efficient heat transport of a wire with a larger radius changes the shape of the critical volume in the same way as an increasing thermal conductivity of metal does.

### Perfusion rates

**Figure 12:**
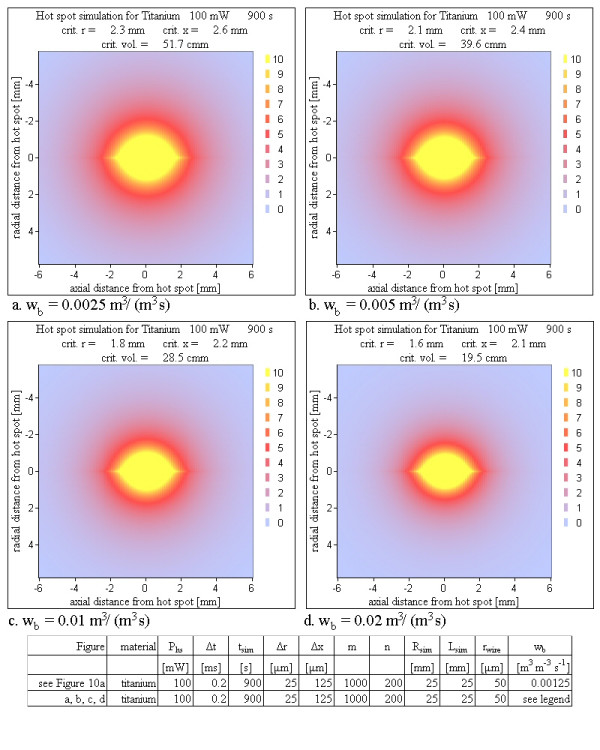
**Various perfusion rates**. The figures show the dependence of the temperature maps on the perfusion rate. Even for a 16 fold perfusion rate compared to the normal perfusion the critical volume is reduced only by a factor of 3.

For all prior described simulations the constant perfusion value corresponding to the normal perfusion value of 0.00125 m^3^/(m^3^s) was used [17]. In the literature blood perfusion is described to increase with increasing temperature [17]. This feature needs not to be considered here, because the effect of cooling by blood perfusion is evaluated here by considering a parameter range for w_b_, which is much broader than the range determined by the temperature dependence. Figure 12 shows the simulation results for increased blood perfusion values. The lowest value used, is the physiological value of w_b _= 0.00125 m^3^/(m^3^s) under normal circumstances (Figure 10a). It is increased up to w_b _= 0.02 m^3^/(m^3^s). The results show the expected decrease in the critical volume, but even the strongest blood perfusion can not suppress the formation of a critical volume.

### Blood flow

**Figure 13:**
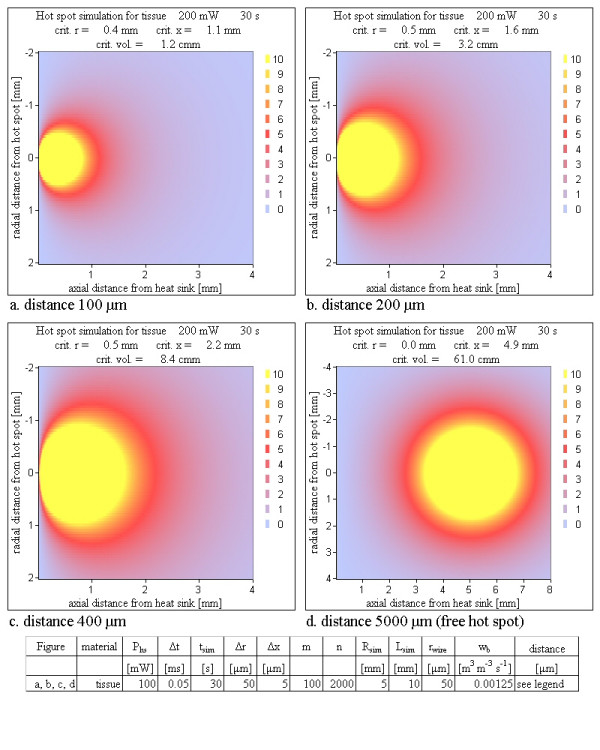
**Various distances between a hot spot and a planar heat sink for a 'tissue only' simulation**. Already within 30 s a hot spot very close to a cooling blood flow – capable of transporting all applied energy immediately away – reaches a substantial critical volume using a critical temperature of 5 K. Notice that Figure 13d is differently scaled. The time development of the critical volume is shown in Figure 14.

**Figure 14:**
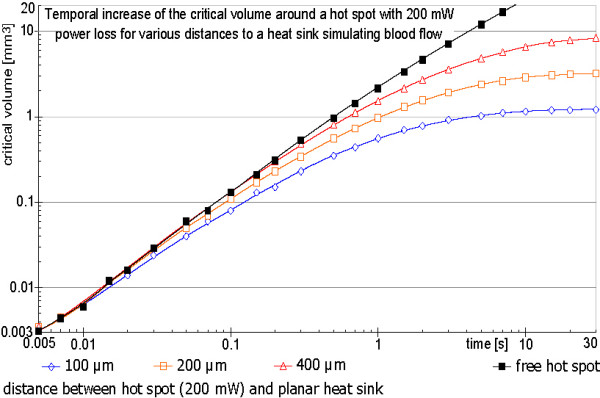
**Temporal development of the critical volume for 200 mW hot spot power loss for various distances between the hot spot and a planar heat sink simulating blood flow**. The maximum critical volume depends on the distance between the hot spot and the heat sink simulating the blood flow inside a large vessel. For a very short distance of only a few cell layers, the critical volume nevertheless reaches more than 1 mm^3^. Increasing the distance strongly increases the critical volume. The four graphs show the fast buildup of the critical volume within a few seconds.

The influence of cooling blood flow was tested by shifting the hot spot near to a planar heat sink as simulation of a hot spot placed inside the wall of a big vessel. Figure 13 shows simulation results for different distances between heat sink and hot spot. Even in the case of a 100 μm distance between the hot spot and the cooling blood flow a large temperature peak at the location of the hot spot and a critical volume of roughly 1 mm^3 ^was calculated for a high power loss. This value of the critical volume is reached within a few seconds (Figure 14).

## Discussion

### Analytical solution

The analytical solution as worst case scenario shows for hot spot power losses above 2 mW first temperature increases above 5 K for a radius near 50 μm (Eq. 3b). Increasing the critical temperature to 10 K, 15 K or 20 K, yields minimal hot spot power losses of about 3 mW, 5 mW and 6. mW respectively. It is important to note, that the analytical solution overestimates the worst case in two parameters. Firstly an MR sequence lasts not long enough to reach the thermal equilibrium. Secondly no metallic wire with a better thermal conductivity is respected, which can distribute the energy of the hot spot more efficient and increases therefore the minimum necessary hot spot power. This is one reason for checking the analytical results with a finite volume analysis.

### Tests of the simulation algorithm

All checks indicate that the algorithm is implemented correctly. The energy inside the simulation volume calculated independently from the simulation algorithm at the end of the calculation, summed with the energy, which has left the simulation volume (heat sinks or perfusion) during the simulated time is equal to the total applied energy W_total _= P_hs_t_sim_. The simulation results also increasingly approach the analytical solution for a sphere inside a homogenous medium for thermal equilibrium using only heat distribution due to thermal conductivity of tissue (Figure 7). Movie 1 (Additional file 1) also indicates that the size of the total simulation volume has been chosen sufficiently large, so that it does not tamper the results.

### Spatial and temporal resolution

Spatial or temporal resolution is of minor importance for a safety check, because they have a weak influence on the critical volume. A high resolution is only necessary, if the steep temperature decay at the metal tissue interface shall be reproduced numerically. For manageable calculation times the spatial resolution is restricted. An example for a good spatial resolution is a matrix of 1000 times 200 and corresponding cell lengths of 25 μm and 125 μm in r- respectively x-direction. The temporal resolution for a titanium wire (r_wire _50 μm) for such a simulation has to be smaller than 750 μs to inhibit oscillatory results. For iron with an fourfold better thermal conductivity the temporal resolution has to be below 300 μs. For this reason all calculations are done with 200 μs. For 900 s simulated time this spatial and temporal resolution requires 4.5 million temperature calculations for each of 200,000 cells. The calculation time using a PC with a 3 GHz processor was more than a day. A resolution twice as good in both directions as the prior described one was only tested once for titanium (Figure 8d). As explained in the results the calculation time is about a week and the results did not differ significantly.

### Time development

Movie 2 (Additional file 2) shows the time development for hot spot between the fracture surfaces of a titanium wire. Even if the simulation calculates the temperature map for 900 s, it can be easily seen that after a few seconds a critical volume appears, which steadily grows during the simulation. For a standard diagnostic MRI investigation a sequence with an SAR of 4W/kg in almost all cases is significantly shorter than 900 s. But a substantial critical volume for a sequence with this SAR is reached within the first seconds even if blood perfusion and blood flow of a very near vessel are considered. Already after one second the critical radius for ΔT = 5 K can reach 0.65 mm corresponding to a critical volume over 1 mm^3^.

### Hot spot power

The hot spot power is of course one of the most important parameters for critical temperature increases. Taking into account an MRI sequence with a maximum SAR of 4 W/kg (manufacturer declaration for the example of table 2) the total power loss density of a resonator can be calculated using Eq. 1 to be 4 mW/cm^3 ^(combined constants of Eq. 1 c_dc_c_pwm_ω_0_B_1_^2^2^-1^μ_0_^-1 ^= 4 mW/cm^3^). The total power loss of a specific resonator can be calculated by multiplying this value with the inductance volume and the quality factor of the resonance circuit inside human tissue. As worst case volume a vena cava filter or a stent graft for an aortic aneurysm with 50 cm^3 ^is assumed with a resonator quality factor of 4 (a quality factor of roughly 3 was experimentally the largest achievable value for resonators with such a large volume inside physiologic saline solution of 0.9% NaCl [11]). This results in a total power loss of 800 mW (Eq. 1, table 2). The hot spot power therefore can reach 200 mW (Eq. 2b). The results of Figure 9 clearly show critical temperature increases for a hot spot power loss of 50 mW. The critical volumes reached are large enough to be dangerous even with additional cooling due to blood perfusion.

### Material

The material of the wire is less important. With all materials substantial critical volumes can be reached (Figure 10). The variations between the critical volumes of different metals are within 20% and critical volumes greater than 1 mm^3 ^are reached in similar times. The maps of Figure 10 show no substantial reduction of the critical volume for different materials.

### Radius

The radius of the metal wire modifies the results more efficiently than the material does, but also an unacceptably large wire radius of 0.5 mm (diameter of 1 mm) reduces the critical volume for a 100 mW hot spot power loss only to roughly one third (Figure 11). The speed of the volume increase is slower for larger radius but nevertheless fast enough to reach critical volumes larger than 1 mm^3 ^in a few seconds.

### Perfusion rate

The model for the simulation of blood perfusion uses no temperature dependence. Instead the constant perfusion parameter is increased over the physiological normal value. The perfusion was increased up to 0.02 m^3^/(m^3^s) (Figure 12). A non-negligible critical volume is reached even for such a 16 fold increased blood exchange rate. The differences calculating the temperature maps with and without blood perfusion can be seen at the end of movie 2 (Additional file 2), which alternately switches at the end between the two simulation models. Adding blood perfusion to a simulation without it makes the simulation a bit less critical, but the cooling by blood perfusion is not sufficient to have safe conditions under all circumstances.

### Blood flow

Starting with a hot spot power of 10 mW, which corresponds to a total power loss of the intact resonator of 40 mW (Eq. 2b), a defect can induce physiologically dangerous heating with starting cell destructions under worst case assumptions. To incorporate the normal anatomical situation of a cooling blood flow, we implement models with a heat sink near the hot spot. This approach is an attempt to model a situation without re-calcification, thrombosis or intima hyperplasia inside the vessel the resonator is implanted to. Such a situation has the cooling blood flow very close to the hot spot and is definitely away from a worse case assumption. The results show that even for this case a physiologically critical temperature increase for a non-negligible volume can be induced. The simulations evaluate a critical volume of more than 1 mm^3^, which is reached within the order of 10 seconds (Figure 14).

Movie 2 (Additional file 2) illustrates the enormous speed of the temperature increase at a small volume within the first seconds. This could lead to immediate tissue damage due to bursting cells adjacent to the hot spot. If the hot spot is located very close to the blood flow such bursts combined with the increased temperature can give raise to a thrombosis inside the vessel near the wall, which shields the cooling blood from the vessel wall and the possible critical volume increase may be enlarged in a vicious circle. Figure 14 shows the situation with the highest assumed cooling (a distance of 100 μm between hot spot and planar heat sink). Even for this short distance corresponding to a few cell layers between the hot spot and the cooling blood flow, the volume can reach 1 mm^3 ^within a few seconds (Figure 14) and temperatures increases over 70 K at the metal wire and directly adjacent cells are reached within a few milliseconds. On one hand the fast rise time may help to reduce the temperature increases, because it can setup an immediate gaseous thermal isolation around the hot spot, which also can change the rupture resistance by an instantaneous volume increase, which for example can disconnect the touching surfaces of a rupture. But this presumption can not be guarantied under worst case conditions. On the other hand with the fast rise time first tissue damages with bursting cells can be reached in a time scale below a second and a developing thrombosis can shield cooling blood flow and increase the reachable critical volume over the values calculated with the simulation.

## Conclusion

The analytical solution as worst case shows, that small active implants with *V*_ind _*Q *< 2 cm^3 ^are definitely safe with respect to any heating due to normal power losses [11,12] or failures. Because inside a living tissue the quality factor hardly exceeds 5, this statement means that resonators with volumes below 0.4 cm^3 ^are safe even under the overestimating worst case scenario. The numerical simulation can calculate maps of temperature increases respecting the metallic wire without reaching thermal equilibrium as well as the additional cooling effects of blood perfusion and blood flow. The results point out, that resonators with *V*_ind _*Q *> 10 cm^3^, corresponding to a smallest inductance volume of 2 cm^3^, can be dangerous. MRI active aortic stent grafts or MRI active vena cava filters with a volume of a few ten cubic centimeters can set up a not negligible critical volume even using conditions far away from the worst case and with additional cooling due to blood perfusion and blood flow. This critical situation can be reached with uselessly low quality factors. For example, an active implant with an inductance volume of 50 cm^3 ^and a quality factor of *Q *= 1 (implanted), which is of no advantage in overcoming any Faraday cage shielding, can reach a hot spot power loss of 50 mW (Eqs. 1, 2b). Such high hot spot power losses reach critical volumes larger than 1 mm^3 ^in a few seconds. The analytical solution as well as the simulations show that the hot spot power is the dominating parameter for the safety investigation. The material or the radius of the wire (stent strut) does not reduce the worst case scenario significantly. Additionally, neither blood perfusion nor blood flow in the direct vicinity of the hot spot reduces the critical volume to an uncritical value as calculated by the numerical results. Considering the fast rise time of the temperatures directly adjacent to the hot spot, bursting cells as well as the increased temperature are very likely to induce a thrombosis shielding the blood flow more and more from the hot spot. A volume of a few cubic millimeters is reached by MR sequences with a high SAR within a few seconds.

For safety reasons, patients with such large active implants should be excluded from MRI investigations at the resonance frequency the resonator is made for. Such large devices are therefore useless in dealing with the Faraday cage effect disturbing the lumen information of metallic implants.

Eventually large active implants can be safely constructed by using additional (passive) electronics, which shortcuts the resonator during the excitations of an MRI sequence and leaves the resonator operational during detection. Such electronics would exclude the flip angle amplification, but maintain the signal amplification during the detection phase.

Another very important safety topic, that should be monitored carefully using active implants, is the achievable amplification homogeneity inside the lumen of such devices. Very low susceptibility artifacts as well as a homogeneous amplification are extremely important for the use of active implants as inductively coupled imaging coils. Although the usual stent materials (for example titanium, Nitinol, stainless steel or tantalum) seem to be non magnetic, they are actually paramagnetic in contrast to the mostly diamagnetic tissue. For small active implants like stents for coronary vessels, which are safe concerning temperature effects, the susceptibility artifacts can inhibit the imaging of a substantial part of the stent lumen. For example a stent with a diameter of 3 mm constructed of a paramagnetic material may seem to have a wall thickness of more than 1 mm in MRI images (depending on the bandwidth of the sequence), although the actual thickness is about 0.1 mm. Such a stent in fact does not benefit from an active technology. A large part of the stent lumen and unfortunately the one near the vessel wall, where critical situations most likely occur, is hidden by susceptibility artifacts for numerous sequences.

For a serious interpretation of the implant lumen a sufficiently homogeneous rf magnetic field inside the resonator coil is also necessary. The well known coil types (solenoid, saddle coil and bird cage) perform quiet well, if they have their ideal geometry. They probably perform worse, if their usual geometry is changed to an expandable version, which is necessary for an implantation by a catheter. An additional distortion of the field homogeneity is unpreventable, if the expansion of the coil by inflation of a balloon is not perfect. As a consequence, the MR sensitivity inside the lumen may vary significantly within a very small region. This can lead to severe misinterpretations of the acquired images. For safety reasons the spatial amplification of active devices has to be investigated depending on not ideal geometries as well as on not perfect expansions. Small active magnetic resonance implants may have a high potential for a non invasive follow up. But before clinical trials numerous unanswered questions must be addressed.

## Supplementary Material

Additional File 1 **Movie 1 of the time developing temperature map for tissue**. This movie (animated GIF) shows the time development for the case of a hot spot in tissue without wire and with blood perfusion over a period of 900 s, which is the maximum permitted time for imaging the trunk with an SAR of 4 W/kg (sequence of table 2, manufacturer declaration of 4 W/kg). For a complete information, a 3D perspective view is shown in figure a. In figure b for a part of the simulation volume all temperature increases above 10 K are replaced just by 10 K to allow a scaling which shows the temperature increases around the hot spot more clearly. In figure c a cross section along the center symmetry axis (the wire) is shown. This plot uses the symmetry to the center axis and the symmetry to the center plane at × = 0 orthogonal to the center axis to show the temperature map of the entire implant, i. e. the total simulation volume. Figure d contains similar information as c. Instead of a 3D perspective view of only tissue temperature increases a map including the wire temperatures coded in colors is shown. At the end of the movie two different simulations are shown alternately. They indicate the changes of the temperature map after 900 s due to a larger simulation volume shifting the heat sink further away from the hot spot. One of the alternating results was calculated using a 250 × 250 matrix for a distance of 0 mm to 12.5 mm for r and × respectively. The second map was calculated for a 500 × 500 matrix for a distance of 0 mm to 25 mm for r and × respectively. Only the inner 250 × 250 points are plotted for a same size for both calculations. It can be seen especially in figure b, that the temperature distribution is almost identical apart from the fact that, for x ≈12.5 mm and r ≈ 12.5 mm, the simulation with more cells shows a slight deviation from zero. The simulation with the smaller matrix shows a straight zero line, which is naturally because this is the boundary condition for this simulation. The small difference points out that the boundary condition with a heat sink works very well as long as the absolute value of the gradient at the boundary is low. Click here for file

Additional File 2 **Movie 2 of the time developing temperature map for titanium wire**. This movie (additional file 2) is very similar to movie 1 (additional file 1). Instead of a tissue only simulation the hot spot now is placed between the two surfaces of a broken titanium wire with 50 μm radius. The tissue temperature increases are shown as 3D perspective view, whereas the temperature of the titanium wire is shown as additional red line. At the end of the movie two different simulations are shown alternately, which indicate the changes of the temperature map after 900 s due to a simulation with and without blood perfusion. It can be seen that the critical volume is reduced with blood perfusion without reaching an uncritical size. Click here for file
