# Supplementary figures and images for: Finite volume analysis of temperature effects induced by active MRI implants: 2. Defects on active MRI implants causing hot spots
Source: Biomed Eng Online. 2006 May 26;5:35. doi: 10.1186/1475-925X-5-35 (PMC1513583; doi:10.1186/1475-925X-5-35)

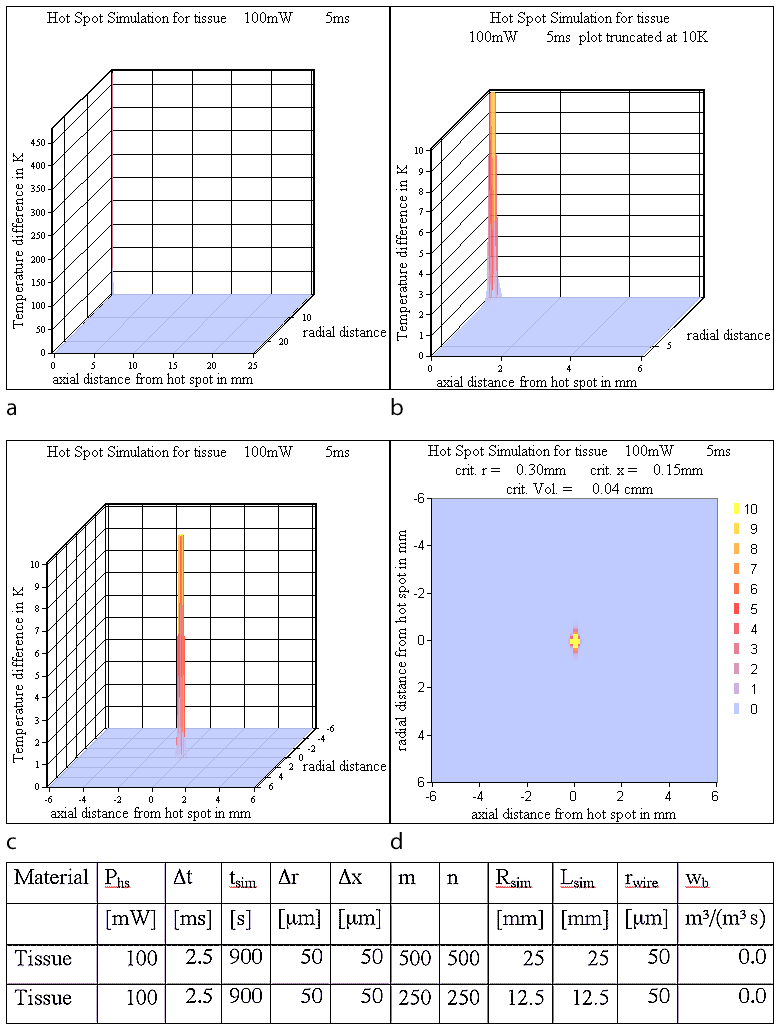

Supplement: Additional File 1 — Movie 1 of the time developing temperature map for tissue. This movie (animated GIF) shows the time development for the case of a hot spot in tissue without wire and with blood perfusion over a period of 900 s, which is the maximum permitted time for imaging the trunk with an SAR of 4 W/kg (sequence of table 2, manufacturer declaration of 4 W/kg). For a complete information, a 3D perspective view is shown in figure a. In figure b for a part of the simulation volume all temperature increases above 10 K are replaced just by 10 K to allow a scaling which shows the temperature increases around the hot spot more clearly. In figure c a cross section along the center symmetry axis (the wire) is shown. This plot uses the symmetry to the center axis and the symmetry to the center plane at × = 0 orthogonal to the center axis to show the temperature map of the entire implant, i. e. the total simulation volume. Figure d contains similar information as c. Instead of a 3D perspective view of only tissue temperature increases a map including the wire temperatures coded in colors is shown. At the end of the movie two different simulations are shown alternately. They indicate the changes of the temperature map after 900 s due to a larger simulation volume shifting the heat sink further away from the hot spot. One of the alternating results was calculated using a 250 × 250 matrix for a distance of 0 mm to 12.5 mm for r and × respectively. The second map was calculated for a 500 × 500 matrix for a distance of 0 mm to 25 mm for r and × respectively. Only the inner 250 × 250 points are plotted for a same size for both calculations. It can be seen especially in figure b, that the temperature distribution is almost identical apart from the fact that, for x ≈12.5 mm and r ≈ 12.5 mm, the simulation with more cells shows a slight deviation from zero. The simulation with the smaller matrix shows a straight zero line, which is naturally because this is the boundary condition for this simulatio [file 1475-925X-5-35-S1.gif]

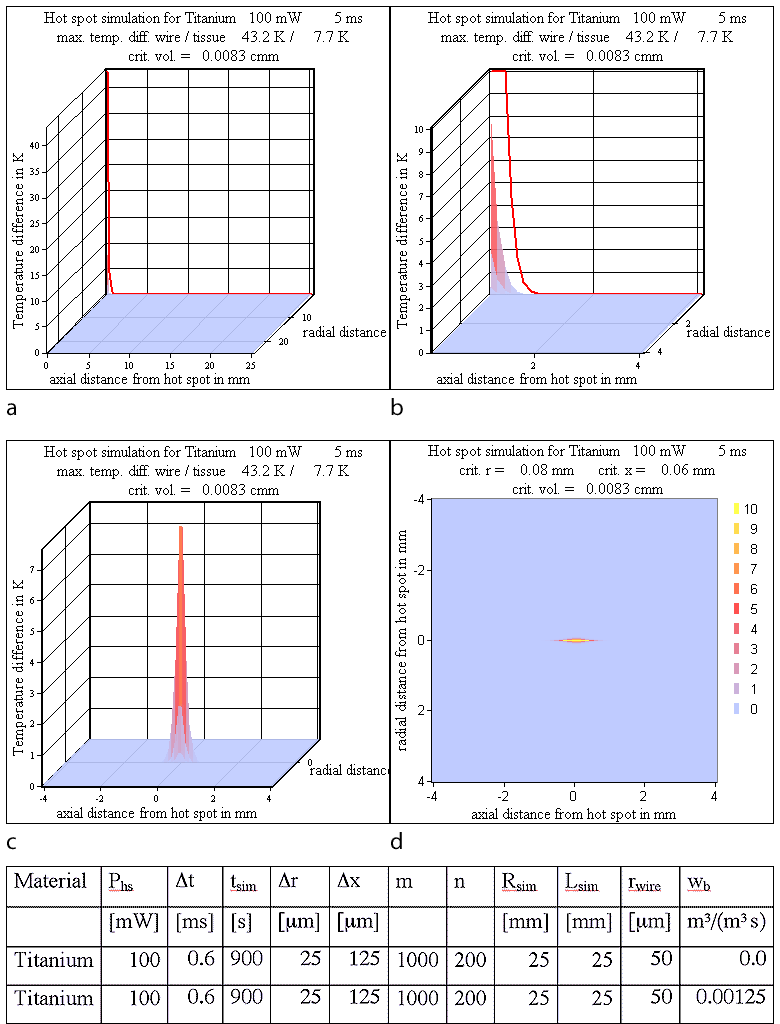

Supplement: Additional File 2 — Movie 2 of the time developing temperature map for titanium wire. This movie (additional file 2) is very similar to movie 1 (additional file 1). Instead of a tissue only simulation the hot spot now is placed between the two surfaces of a broken titanium wire with 50 μm radius. The tissue temperature increases are shown as 3D perspective view, whereas the temperature of the titanium wire is shown as additional red line. At the end of the movie two different simulations are shown alternately, which indicate the changes of the temperature map after 900 s due to a simulation with and without blood perfusion. It can be seen that the critical volume is reduced with blood perfusion without reaching an uncritical size. [file 1475-925X-5-35-S2.gif]
